# Role of community engagement in advancing vaccine equity

**DOI:** 10.3389/fpubh.2024.1435231

**Published:** 2024-09-20

**Authors:** Samantha Smith, Erika Marquez, Amanda Haboush-Deloye, Tiana Tu, Aaliyah Goodie, David Perez

**Affiliations:** ^1^Nevada Institute for Children’s Research and Policy, University of Nevada, Las Vegas, Las Vegas, NV, United States; ^2^Department of Environmental and Occupational Health, University of Nevada, Las Vegas, Las Vegas, NV, United States; ^3^Division of Public and Behavioral Health, Nevada Department of Health and Human Services, Carson City, NV, United States

**Keywords:** community-engaged, vaccine equity, partnerships, community collaborations, vaccine confidence, COVID-19, conceptual framework

## Abstract

The COVID-19 pandemic exacerbated existing health disparities among historically and currently underserved, underresourced, and marginalized communities worldwide. These communities faced disproportionate COVID-19 morbidity and mortality and were generally less likely to receive a COVID-19 vaccine once it became widely available to the public. Community engagement is an approach that can help bridge these inequities. This community case study adapted and implemented an existing community engagement framework to tailor a statewide vaccine equity effort that addresses community-specific priorities during a public health emergency. The adapted framework includes the following key phases: (1) creating an environment for community engagement; (2) making the work relevant; (3) narrowing the focus; (4) planning and conducting the work; and (5) evaluating the work. All of these supported the successful establishment of a statewide collaboration that consisted of various partners from various sectors who shared a collective commitment to increase COVID-19 vaccine confidence and address barriers to vaccination among the diverse communities in Nevada. Ultimately, a community engagement framework can provide a roadmap to navigate the dynamic and multifaceted nature of equity-related work by paving the way for meaningful interventions to mitigate health disparities.

## 1 Introduction

Historically and currently underserved, under-resourced, and marginalized communities suffer from poorer overall health outcomes; these disparities have been further demonstrated by the health impacts of the COVID-19 pandemic (1, 2). Throughout the pandemic, racial and ethnic communities, including African American/Black, Hispanic/Latino, American Indian and Alaskan Native individuals, carried a disproportionately higher burden of infections, hospitalizations, and deaths compared to non-Hispanic White individuals (1, 3–5). Many of these communities encounter social and economic challenges as a result of systemic and structural racism that hinder access to resources that support their health and well-being which contributes to an increased risk of adverse health outcomes. However, despite these inequities and injustices, many community groups, nonprofits, and local agencies across the nation have collaborated to develop and implement community-driven efforts that successfully address the specific systemic or structural challenges they face.

With the COVID-19 pandemic, it was particularly imperative to address the ever prevalent and deeply persistent health disparities, especially in light of the United States’ growing diversity (6, 7), with community-driven efforts that are culturally and locally-based. As the COVID-19 vaccines became available in the United States in late 2020 and early 2021, many of the communities that experienced disproportionately higher rates of COVID-19 morbidities and mortality tended to have lower vaccine coverage compared to other racial and ethnic groups (8, 9). By the end of April 2021, Hispanic, non-Hispanic African American/Black, and American Indian and Alaskan Native individuals across the United States had lower rates of at least one dose of COVID-19 vaccine coverage (47.3, 46.3, and 38.7%, respectively) compared to Asians (69.6%) and non-Hispanic White individuals (59.0%) (8). Numerous community groups, nonprofits, and state and local governments, including the state of Nevada, responded to the concerning data on COVID-19 vaccine coverage by launching community-engaged efforts aimed at ameliorating these coverage gaps.

Community engagement (CE) is the process of collaborating with groups of people to address issues that impact their health and well-being, and is an essential component of public health that can bridge gaps and advance health equity (10, 11). CE tailors interventions around the diversity of communities to address the unique factors that contribute to inequities in their health outcomes by building trust and new resources, improving communication, and advancing public health action (10, 12). Despite structural inequities, community-driven groups and community-academic partnerships sprung into action to mitigate the impacts of COVID-19 in their communities. Unidos en Salud, an academic/community partnership, is just one example that demonstrates how a local and culturally based CE approach can help overcome barriers to vaccination in the local community (13). In February 2021, in response to the disproportionate distribution of COVID-19 vaccines in Nevada, Governor Steve Sisolak called for an intentional effort for equity and fairness among those who have been disproportionately impacted by COVID-19 (14). As a result, Immunize Nevada and the Nevada Minority Health and Equity Coalition, two organizations with histories of instituting CE across the state, formed the Nevada Vaccine Equity Collaborative (NVEC) to support statewide COVID-19 vaccine equity efforts. CE was central to NVEC’s focus and commitment to working with Nevada communities that have been historically underserved, under-resourced, and marginalized.

Given the challenges of the COVID-19 pandemic and the probability of future public health emergencies, preparation to implement contextually and culturally informed responses that ensure fair access to resources, programs, and services to safeguard the well-being of all communities is critical. There is a growing body of work that supports CE efforts to address community priorities and the social determinants of health that lead to inequitable health outcomes. This community case study draws upon an established CE model and adapts it to illustrate how to utilize and implement a CE framework to address community priorities during a public health emergency.

## 2 Background of the NVEC

Nevada is the sixth fastest-growing and among the top 10 most racially and ethnically diverse states in the United States (7, 15). Similar to patterns seen across the nation, Nevadan communities of color and populations historically excluded experienced disproportionate COVID-19 morbidities and mortality (16). Once the COVID-19 vaccines became available, data reports revealed inequities in the vaccine distribution process. More specifically, communities disproportionately impacted by the pandemic were the least likely to vaccinate in early 2021 (5, 14). As a result, Immunize Nevada and the Nevada Minority Health and Equity Coalition co-created NVEC to support COVID-19 vaccine equity efforts by increasing vaccine confidence and addressing barriers to vaccination among Nevada’s diverse communities. NVEC committed to applying a CE approach and identified three key aims to support their efforts: (1) to create a hub to facilitate statewide collaboration on COVID-19 vaccine efforts by optimizing partnerships, resources, and opportunities; (2) to provide data-driven recommendations about where to prioritize COVID-19 vaccine distribution based on the CDC/ATSDR Social Vulnerability Index (CDC-SVI) and vaccine coverage rates by zip code tabulation area (ZCTA); and (3) to create culturally and linguistically responsive COVID-19-related educational materials that were relevant to Nevada’s diverse communities to increase vaccine confidence and uptake.

## 3 Framework for community engagement and its application

CE can foster health equity by creating a space for communities to have a “seat at the table” to voice their concerns on matters impacting their overall health and well-being. Implementing CE can result in tailored initiatives or interventions that appropriately reflect the diversity and needs of a community. Several CE frameworks provide a structure and set of principles to help guide the interactions and methods of collaboration between diverse partnerships. Given the many available frameworks, it is evident CE is not a simple nor linear process; it is both a science and art, which can take on many structures depending on the context and dynamics of the groups that are working together (10). CE functions on a continuum based on the level of participation from the community and can take on the form of a partnership, collaboration, or coalition (10). Regardless of the structure, a common component for all forms of CE is participation from diverse groups and disciplines.

**Figure 1:**
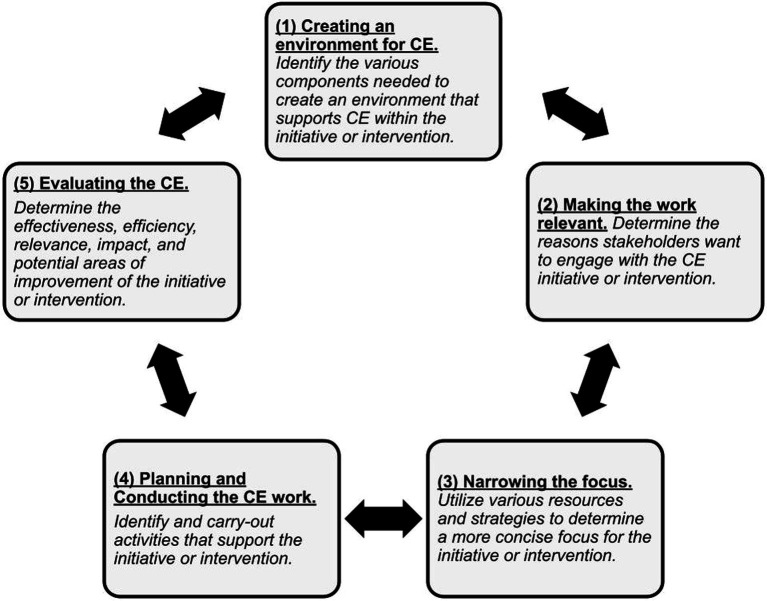
Conceptual CE framework for NVEC.

The *Community Engagement in Interventions: Conceptual Framework* outlines several components necessary for an effective, sustainable, and appropriate public health initiative that uses CE, which are ways of defining communities and health needs; initial motivations for CE; types of participation; conditions and actions necessary for engagement; and potential issues influencing impact” (16). This framework encourages a fit-for-purpose approach to assist public health professionals, researchers, and community members in examining the philosophical underpinnings of interventions they employ. In turn, NVEC utilized and adapted components of this CE framework into a new and unique conceptual framework to guide its vaccine equity efforts (Figure 1).

This community case study walks through each phase of the adapted CE framework, providing an overview of each phase and its application within NVEC.

### 3.1 Creating an environment for CE

The first phase in the adapted framework is to determine the various components needed to create the structure and an environment that supports CE within the initiative or intervention. These components could include staffing, funding, partnerships, skills, expertise, shared values, training, supplies, and physical space.

Within the first 2 weeks of February 2021, Immunize Nevada and the Nevada Minority Health and Equity Coalition formed an NVEC leadership team composed of staff members from each organization to handle the internal operations of the collaborative. Their responsibilities included facilitating regularly scheduled statewide meetings, recruiting partners to join the collaborative, producing vaccine maps, and coordinating pop-up vaccine clinics. The team held meetings via Zoom to allow statewide participation while adhering to state-recommended social distancing guidelines. Lastly, both lead organizations were able to secure funding that supported their and community partner organizations’ vaccine equity activities.

Then, the leadership team developed a set of core values and principles to guide NVEC’s CE efforts, which derived from input and feedback from coalition members of the Nevada Minority Health and Equity Coalition (Table 1). These values and principles are fundamentally rooted in coalition-building work and created a framework to build trust, guide decision-making, promote transparency and accountability, facilitate effective communication, and promote cultural sensitivity within NVEC. To implement these values and principles, the leadership team recruited over 200 partners from various sectors and disciplines who have diverse expertise, skills, and a shared goal of promoting equitable distribution of COVID-19 resources and vaccines throughout Nevada. NVEC’s diverse partners included community-based organizations, faith-based leaders, healthcare providers, county and local health district representatives, state legislators, pharmaceutical representatives, public health researchers, community health workers, students, and community members.

**Table 1:** NVEC core values and principles.

Value	Principles
Equity	Share decision-making and leadership Adapt support strategies as necessary to ensure fair treatment and outcomes Treat participants with integrity and respect
Inclusivity	Provide equal access to opportunities and resources Strive for community representation and inclusion of groups often underrepresented Create a space in which communities feel valued and welcomed
Diversity	Engage community members with different backgrounds, beliefs, and experiences, such as race/ethnicity, citizenship status, religious beliefs, socioeconomic status, language, geographical origin, gender and/or sexual orientation Recognize differences are assets to learning and innovation
Cultural humility	Maintain awareness of power imbalances and biases, respect other’s values, and do not set personal expectations to memorize all aspects of another culture Understand how personal biases may impact your work Continuous self-reflection to examine own beliefs and cultural identities
Accountability	Build processes that are responsive to feedback from community partners Willingness to change, pivot, and adapt throughout the process
Transparency	Communicate openly about motives, resources, power dynamics, and decision-making processes Openly acknowledge challenges and limitations in order to maintain trust
Sustainability	Continually reflect, assess, and communicate to maintain and deepen relationships for long-term action Allocate adequate resources to maintain relationships with communities
Capacity building	Support existing community leaders and develop new leaders Increase community involvement, impact, trust, and communication by improving coordination, enhancing existing services, advocating for policy change and learning through pilots

Adapted from the New York City Department of Health and Mental Hygiene (17). Race to Justice: Community Engagement Framework. NYC Health.

To create an environment that upholds NVEC’s values and principles, the leadership team hosted meetings that were a hub for partners to receive COVID-19-related updates, build interdisciplinary relationships, and collaborate on the development of educational messaging materials and vaccine strategies. These meetings included time for open and honest discussions, where partners could share successes, challenges, and opportunities related to their COVID-19 vaccine efforts within communities across the state. Meetings also served as a way to collectively brainstorm solutions to identified challenges and collaborate on upcoming events. Beyond the meetings, the NVEC leadership team was always available to partners to provide technical as well as general support for their specific vaccine equity efforts. NVEC leadership recognized a commitment to providing ongoing support, especially during the first year of vaccine roll-out, was critical and would occasionally require availability outside of regular work hours and week. Ultimately, NVEC structured its approach to continuously foster CE opportunities where partners could collaborate and engage at a level they found most comfortable. For instance, some partners preferred a less involved role by only attending meetings to receive COVID-19-related updates and resources. In contrast, other partners took on more involved roles by providing recommendations on messaging materials or offering their location as a pop-up vaccination site.

### 3.2 Making the work relevant

The second phase of the CE framework is about making the work relevant. In other words, it relates to the motivations or reasons community members, partners, organizations, or other key stakeholders would want to start a new initiative or intervention and engage with it. These motivations can be intrinsic (e.g., wanting to develop a skill, gain knowledge, or improve personal health and well-being) or extrinsic (e.g., wanting to achieve a common goal, address a shared priority, advance scientific knowledge, improve the community environment on a physical, social, or economic level, or access funding that is currently available).

For NVEC, four key factors drove its development and necessity: (1) the COVID-19 pandemic amplified existing health disparities and inequities throughout the state, (2) data revealed that communities disproportionately impacted by the pandemic were the least likely to get vaccinated once the COVID-19 vaccines were available, (3) the Nevada state governor called-to-action a task force to work toward COVID-19 vaccine equity, and (4) there was funding readily available to support this type of effort. The combination of these factors suggested the need for an effective statewide response to reach communities that were underserved and under-resourced during the pandemic, which required community input and interdisciplinary expertise.

The establishment of NVEC was both timely and relevant to the work of various stakeholders in Nevada. A combination of motivations drove these stakeholders to join NVEC. For example, community members and organizations highly embedded in the communities they live and work in drove to reduce the impacts of COVID-19. At the same time, some, who may not be as well connected, wanted to offer their skills, expertise, assets, and resources to positively impact those communities. Others were interested in bridging gaps in access to COVID-19 resources and services, establishing new partnerships with other organizations and individuals, staying up-to-date on pandemic-related information, learning best practices for delivering COVID-19 resources and services to diverse communities, and supporting efforts to determine where to prioritize efforts. While each partner’s individual motivations to sit at the table and join NVEC may have differed, the concept of COVID-19 vaccine equity was shared among all partners.

### 3.3 Narrowing the focus

The next phase in the adapted framework is narrowing the focus of the initiative or intervention. There are a variety of techniques to narrow the focus of the work, such as reviewing current scientific literature and available data, conducting a survey, or engaging in community conversations. A combination of these strategies is needed to determine which communities are impacted most, their priorities, and the related social determinants. It is also particularly important to consider individual and collective capacities at this phase since the availability of resources will likely drive the direction of the effort.

There are two parts to this phase. The initial step is identifying communities that find this work relevant to their community needs and those that are impacted the most. Communities are defined as groups that share at least one common attribute, such as geographic location, culture, interest, value, and social or economic characteristics (18). The second step in this phase is determining the priority issues of the impacted communities and the associated determinants. Priority issues describe a concern that impacts the health of the community, while the associated determinants describe conditions that influence those health impacts. Issues and determinants can be identified by the community itself, observations from external parties, a review of currently available data, or comparisons with similar communities (18). Ultimately, clearly defining the communities, issues, and determinants related to the initiative or intervention will narrow the focus and allow the group to develop actionable steps and a clear path to achieve its expected impacts.

NVEC first identified communities most impacted by the pandemic by reviewing available data on COVID-19 infection and vaccination rates at the time. NVEC used two methods to identify priority communities. First, empirical data using COVID-19 infection and vaccination rates paired with the CDC-SVI to determine the location of the most impacted communities (e.g., those with low vaccination and high social vulnerability) at a given time. Second, NVEC relied on community partner input to provide information that may not have been captured by the available data. More specifically, NVEC was able to offer some funding through sub-grants to support community partners’ efforts and also maintained flexible communication channels (i.e., Zoom meetings or phone calls outside of regular business hours or in-person meetings at convenient community locations) where community partners could share their on-the-ground insights about factors driving higher rates of infection and lower rates of vaccination in their communities. For instance, in some communities, culturally and linguistically relevant resources communicating COVID-19-related information was scarce. In other communities, it was a matter of access to a COVID-19 vaccination site that met their needs (e.g., at a familiar location in the community, staff that reflected their community, materials that were readily available in their language, and no specific ID requirements). These two methods were employed to determine the priority issues and associated determinants driving the disproportionate COVID-19 impacts within these communities.

### 3.4 Planning and conducting CE work

The next phase in the framework is to plan and then conduct the CE activities. These activities will involve the community and address previously identified priorities and associated determinants. The first part of this phase is to plan the CE activities based on the desired changes or impacts (Table 2). For example, one of the community’s priorities could suggest improving an existing community-based system or the physical environment. Thus, the plan should include the resources needed, activities that should be undertaken to accomplish the desired change or impact within the system or environment, how to measure the change, and who is responsible for each step. Ultimately, the plan is to ensure fidelity, flexibility, and functionality within the proposed CE activities. After establishing a plan, the next step is to carry out and implement it.

**Table 2:** Strategies to affect change.

Category	Strategy
*Advancement in Science*	Increasing the understanding of the root causes of health disparities Improving approaches to address health disparities Setting the stage for future research.
*Environment*	Improving elements in the physical, social, and economic environment.
*Policy*	Implementing a new policy, which could be a formal or informal law, ordinance resolution, mandate, or regulation. Revising an existing policy. Advocating for a new policy or the revision of an existing policy.
*Practices*	Improving existing practices within an organization or community, such as day-to-day procedures or standards of operation. Establishing a new practice within an organization or community.
*Systems*	Developing a new program or service to address health disparities in the community. Improving an existing program or service which could include increasing capacity, staffing, and financial resources.

The planning of NVEC included many activities, which were informed either by COVID-19 data, input from on-the-ground partners, discussions with members from the impacted communities, or various combinations of each of these modes. NVEC’s activities included providing sub-grants to community partners to support their on-the-ground vaccine equity efforts, developing a communication and dissemination model that could guide statewide vaccine equity efforts called *Approaches to Vaccine Equity*, providing data- and community-informed recommendations to organizations that are leading COVID mitigation and vaccination efforts across the state, developing culturally and linguistically responsive COVID-19 resources, conducting outreach and education within impacted communities, addressing policies that created barriers to access vaccines, and planning pop-up vaccine clinics in impacted communities. Additionally, NVEC provided a forum to gain new insights, strategies, and skills that supported building the capacity of partners and their respective organizations to address COVID-19-related disparities and inequities.

#### 3.4.1 Example of a policy change driven by CE

A funded community partner who works with migrants who may be undocumented was interested in hosting a pop-up clinic but expressed concerns to NVEC leadership about the enforcement of an ID requirement to receive COVID-19 vaccines at clinics across the state, including pharmacies and larger federal- or state-run sites. More specifically, members from this community were concerned about identifying themselves at a federal- or state-government-run clinic. NVEC leadership shared these concerns directly with state leadership and was able to modify this requirement for community partner-led pop-up clinics where they did not have to enforce it. Subsequently, NVEC’s community partner hosted a pop-up clinic at their trusted location in the community that was primarily staffed by volunteers who reflect and speak the language of the community. This policy change drove the success of this clinic, and future community pop-up clinics across the state, by addressing a community-specific barrier, providing a vaccination opportunity for those who were ready-and-willing, and resulting in several same-day word-of-mouth referrals.

### 3.5 Evaluating CE work

The last phase of this framework is to evaluate the CE efforts. Evaluation is a systematic way to determine the effectiveness, efficiency, relevance, and impact, as well as identify potential areas for improvement of a particular effort (19, 20). It can occur during development (formative evaluation) and implementation (summative evaluation) (19, 20). To conduct an effective and meaningful evaluation, it is highly recommended to establish a combination of clear goals, objectives, measures, and baselines to serve as a point of comparison.

After establishing NVEC in February 2021, internal staff periodically conducted a process evaluation to determine whether activities were implemented as intended. The team tracked the number of partners, meetings, meeting attendees, newsletters, data reports with maps, materials, and resources created and delivered, NVEC-hosted events, and other pertinent metrics to show who was reached, what was done, and when it happened. Since the NVEC’s establishment in February 2021 to March 2024, it has hosted 34 statewide meetings, distributed 28 newsletters, developed 49 vaccine data reports with CDC-SVI maps, created a vaccine equity toolkit, hosted six telephone town halls, and presented four breakout sessions and one poster session at professional conferences. Ongoing evaluation allowed NVEC staff to assess whether there were any particular barriers or facilitators related to these activities, allowing the team and partners to strategize on how to effectively meet our goals. For example, NVEC initially hosted a general meeting and two separate work group meetings on a regular basis. However, once the vaccines were more readily available to the general public, the individual workgroup meetings became less productive and were not as well attended as the general meetings. Therefore, NVEC staff integrated the activities of the two workgroups into the general meeting and dissipated the workgroups.

In addition to the process evaluation, NVEC staff conducted an ongoing outcome evaluation to determine its effects on increasing COVID-19 vaccine access and acceptance among communities that were disproportionately impacted by the pandemic. NVEC evaluated this outcome based on the number of vaccination clinics held in zip codes with high CDC-SVI and low vaccine uptake and the percentage of completed COVID-19 vaccination series among populations living in high CDC-SVI zip codes. In collaboration with community partners, more than 300 vaccine pop-up clinics were hosted in high CDC-SVI and low vaccine coverage areas throughout 2021. In March 2021, about 9% of the population living in high CDC-SVI zip codes had completed a COVID-19 vaccination series, compared to the 15 and 19% in moderate and low CDC-SVI zip codes, respectively. Each month, roughly 40% of the completed vaccinations occurred in high CDC-SVI zip codes. By the end of December 2021, nearly 51% of the population living in high CDC-SVI zip codes completed a COVID-19 vaccination series. Overall, the vaccine pop-up clinics contributed to the nearly 467% increase in completed COVID-19 vaccine series in high CDC-SVI zip codes in 2021, whereas the moderate and low CDC-SVI zip codes had only a 270 and 227%, respectively, increase in completed vaccinations.

## 4 Discussion

As health disparities and inequities continue persisting and widening among communities that are economically and socially marginalized, there is a need to adopt CE approaches when guiding health equity efforts. CE frameworks provide a dynamic roadmap for navigating the multifaceted nature of health equity work and addressing systemic and structural issues at their root cause. This community case study demonstrated how leveraging and implementing a CE framework helps tailor a health equity effort amid a public health emergency to yield effective and relevant strategies and measurable outcomes (21–26).

Consistent with the literature (27–29), NVEC highlights the importance of community-driven efforts to address community-specific priorities during a public health emergency. NVEC’s strategic partnerships increased the availability of culturally relevant, fact-based, and responsive information related to COVID-19 and addressed barriers to COVID-19 vaccinations among communities most impacted by the pandemic. The collaborative also showed outcomes that were not as easily quantified. For instance, the present initiative created a “seat at the table” for community partners who did not regularly interact with local, state, and federal governments and vice versa. Creating an environment for CE produced opportunities to develop new partnerships and collaborations beyond vaccine equity work. This community case study is consistent with the existing literature that supports the notion that CE work offers opportunities to engage, sustain, and maintain authentic relationships that support community buy-in when the community is integrated into the Decision-making process about what and how things should be done (21, 27–32). Vaccinations are one of the most cost-effective interventions that can be employed to prevent and mitigate the impact of communicable diseases. A meta-analysis examining the impact of CE interventions to improve childhood immunizations in low- and middle-income countries found that CE has “a small but significant positive effect on all primary immunization outcomes related to coverage and timeliness” (33). Another study found implementation of community-informed communication strategies increased childhood immunizations from 45% at baseline to 82% over a 4-year period (34). Although community-engaged work is effective in improving health, there is no one-size-fits-all model (35).

As with any approach, the implementation process provides valuable insights and lessons learned. Some key takeaways from NVEC when engaging in CE work involve relationship building, context, collaborative decision-making, flexibility and adaptability, and impact and action. *Relationship building* is essential to effective CE and requires building trust between partners, which takes significant time, effort, and genuine engagement. An emergency response during a pandemic may not seem like the most conducive time to undertake CE work. However, NVEC served as a hub to support interdisciplinary partnerships across the state to strengthen the pandemic response. Many of the partnerships within NVEC were formed as part of the emergency response, while others were partners brought into the initiative from past collaborations. Despite the challenging time to build trust among new partnerships, the relevance of the issue, NVEC’s sincere commitment to integrate the community into the response (e.g., creating a seat at the table), and the ability to fund partners for their time and effort forged the foundation to build new partnerships. Additionally, when “creating a space” for partners, it is more than a metaphoric analogy. It means being prepared to have tough conversations that may challenge what one would consider evidence-based practices and adopt out-of-the-box strategies. It goes without mentioning that building relationships sometimes entails conversations, texts, and emails outside traditional business hours.

As researchers and community members, a commitment to integrating *context* into the development of intervention strategies is important because each community will have a unique social, cultural, economic, and political system in which they live, work, play, and worship. Thus, it is imperative to develop and implement interventions that are respectful and sensitive to these dynamics. A one-size-fits-all model does not integrate the principles of CE. This requires listening to the unique challenges and needs of the community. In this case study, it meant adapting communication strategies for communities based on real-time concerns and questions. *Collaborative decision-making* is integral. Therefore, it is important to be aware of and think about how to navigate power dynamics. Power imbalances do not foster collaborative and inclusive environments. Creating a “seat at the table” entails respect for local knowledge and the valuable contributions and expertise that knowledge brings to understanding the inequities and the determinants that continue to plague health outcomes in these communities. Input from on-the-ground community partners and members provided insights into the realities and challenges of vaccine implementation. For instance, some partners revealed acute awareness of vaccination challenges and hesitancies among our undocumented communities under initial implementation strategies. If it were not for the knowledge they brought to the table and the potential solutions, there likely would have been fewer undocumented community members vaccinated.

*Flexibility and adaptability* are core principles of CE work. Part of the nature of CE, especially in the face of a pandemic, calls for flexibility and adaptability amongst a variety of methods that are contextually appropriate. No single solution will work in every scenario, and out-of-the-box thinking was highly encouraged as dissemination and vaccination efforts were pushed through communities. As the landscape of the pandemic evolved, maintaining a willingness to learn and adapt based on the changing circumstances fostered an environment for NVEC to maintain effective and diverse methods of CE. This also required being reflexive to community input and adapting actions tailored to that input. Actions vary depending on contextual factors and other elements unique to each community. Multiple methods for CE were employed as well, such as participation in community outreach events with NVEC partners. The *impact and action* of CE work fosters solutions and sustains relationships. Public health efforts must carefully develop and integrate CE work while capturing and tracking both process and outcome measures to assess the work’s impact and guide the informed development and actionable strategies. It is crucial to evaluate efforts on an ongoing basis during implementation. It allows for the re-prioritization of efforts based on the current data and the identification of barriers and facilitators to reaching intended outcomes. CE is fluid, and a successful practice may see that the needs and priorities of partnerships evolve throughout the process.

Community-engaged approaches have been used in various fields of work to solve complex problems (36). CE has been instrumental in bringing meaningful and impactful change in communities (30). NVEC’s work toward vaccine equity in the face of COVID-19 highlights tremendous value in CE among Nevada’s at-risk populations, those being communities who face socio-economic barriers and are historically marginalized and, therefore, are at greater risk of adverse health outcomes. Vaccine equity is only one facet of health equity. Health equity is dynamic, with many components to consider when working among communities with unique perspectives, experiences, and concerns. Future public health research and interventions in community-based settings should develop actions that put community priorities and culture at the forefront of decision-making to bridge gaps in health equity. Endeavors should not merely focus on health equity as an outcome but as part of the process of CE. Community-engaged approaches centered around equity will ensure meaningful responses when working with those burdened by health disparities.

## Data Availability

The original contributions presented in the study are included in the article/supplementary material, further inquiries can be directed to the corresponding author.
